# Board Invited Review: Crossbreeding beef × dairy cattle for the modern beef production system

**DOI:** 10.1093/tas/txac025

**Published:** 2022-02-09

**Authors:** Bailey L Basiel, Tara L Felix

**Affiliations:** Department of Animal Science, Pennsylvania State University, University Park, PA 16802, USA

**Keywords:** beef, breeding, crossbred, dairy, sire selection

## Abstract

Current trends in the United States dairy industry suggest that crossbred beef × dairy calves are replacing a proportion of the calf-fed Holstein steers slaughtered for beef each year. Economic pressures value preweaned beef × dairy calves at a premium over preweaned dairy bull calves; however, there is little modern data to support that intensively fed crossbred calves maintain their premium value over dairy steers across the supply chain. Data from international production systems and from historic research suggests that beef × dairy cattle had greater average daily gains and converted feed to gain more efficiently than dairy steers. Regarding carcass characteristics, across the literature crossbreds consistently yielded heavier carcasses that had lower proportions of trim than dairy steers. Fewer comparisons of beef × dairy and dairy steers exist in the literature for other economically relevant carcass characteristics such as ribeye area, backfat, marbling, tenderness, and eating quality. Existing published data are inconsistent among studies, highlighting the necessity for more research tailored to the United States beef production system

## INTRODUCTION

With the drought of 2012, and the subsequent recession in beef cow numbers in the United States, the beef industry began to explore alternatives to maintain beef supply. One such alternative included increased sourcing of dairy-type animals to supply beef. From 2011 to 2016 the proportion of dairy-type carcasses in the supply chain increased from 9.9% to 16.3% (Boykin et al., 2017). What followed, was a rapid growth in the, heretofore slim (Thonney et al., 1987; Tjardes et al., 2002), body of scientific literature available about proper nutrition and management strategies to improve upon the Holstein genetics to yield a viable beef production model, known as the calf-fed Holstein model (Carrasco et al., 2013; Martin et al., 2014; Carvalho and Felix, 2021; Carvalho et al., 2021). The calf-fed Holstein model was recently reviewed by Schaefer et al. (2017).

**Figure 1.**
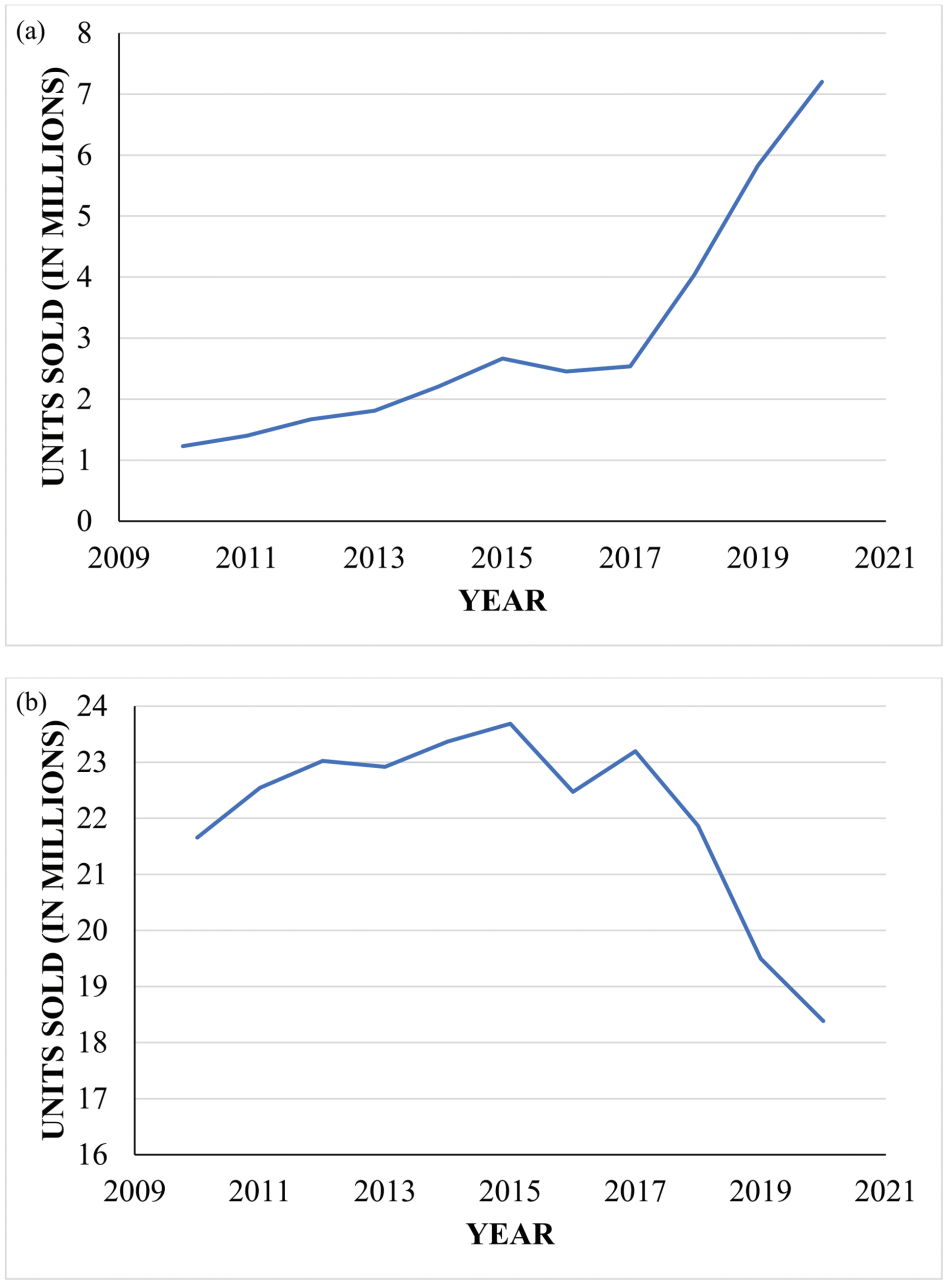
Domestic (a) beef and (b) dairy semen sales in the United States over the last decade.

In 2016, when native beef supply began to return and normalize, the Holstein bull calf lost almost all value. The value of dairy heifer calves plummeted 2 years earlier in response to poor milk prices and had not yet recovered. These 2 events left dairy farms without a market for byproduct male calves or excess female replacements. To add value to surplus calves, more dairies began mating a portion of their females to beef semen. These trends are reflected in the over 260% increase in domestic beef semen sales from 2017 to 2021 and the subsequent reduction in domestic dairy semen sales (NAAB, 2021; Figure 1). The increase in beef semen sales has been largely attributed to the dairy industry because over 90% of beef females in the United States are mated exclusively by natural service (USDA, 2020).

The beef × dairy calf resulting from these matings is currently more valuable shortly after birth to buyers than the dairy bull calf (Dal Zotto et al., 2009; Wilson et al., 2020). However, market variability and downstream packer logistics have somewhat limited the value of crossbred beef × dairy calves to feedlots, largely because their acceptance at packing plants remains variable. In some cases, packer pricing structures are still in line with the pricing structures for purebred Holstein steers.

Modern data on growth and terminal performance of beef × dairy cattle reared in the US beef industry are limited, especially in comparison to the growing body of literature on the calf-fed Holstein. However, historically, and internationally, beef × dairy cattle production has been investigated and reviewed many times (Carter, 1975; Shanks, 2003). Most recently, Berry (2021) provided a comprehensive review of global beef × dairy literature in respect to generating and rearing crossbred calves in a variety of production systems.

This review will examine the existing literature on beef × dairy cattle through the lens of modern beef production in the United States. It will explore the economic conditions that have motivated the reemergence of beef × dairy and examine the current trends in beef semen usage on US dairies. Economically relevant performance traits of beef × dairy calves will be compared to that of purebred dairy calves and sire selection, regarding such traits, will be discussed. In all instances where crosses are referenced, sire breed will be referenced first, followed by dam breed in a sire × dam format.

## BEEF × DAIRY ECONOMICS IN THE DAIRY HERD

The use of artificial insemination in the dairy industry is commonplace. As such, genetic progress can be accelerated through selective matings. Young dairy females are genetically superior to older females in the herd (CDCB, 2021). Dairy herds are able to advance genetic progress by generating replacement females from their genetically superior animals, which affords a group of older cows with less genetic potential that can be mated for other purposes. Beef sires have provided an opportunity to add value to dairy bull calves that can, at times, be a net loss byproduct of dairy operations. When combined with sexed dairy semen, the incorporation of beef semen in the dairy herd has economic and genetic advantages. This practice ensures replacements from the genetically superior animals in the herd and generates value-added beef × dairy progeny from the remaining females; thus, profitability is maximized in a model that includes selective use of sexed dairy semen and conventional beef semen (Ettema et al., 2017; Pahmeyer and Britz, 2020; De Vries et al., 2020; Clasen et al., 2021).

International data suggests that beef × dairy crossbred calves add value to dairy operations. When considering the gross value of crossbred calves, an analysis of auction records in a region of Italy reported that beef × dairy calves were valued 50%–200% more per kg than purebred Holstein or Brown Swiss calves (Dal Zotto et al., 2009). An analysis of Canadian auction records stated that, on average, beef × dairy bull calves sold for $30 CAD, $92 CAD, and $140 CAD more than Holstein, Brown Swiss, and Jersey bull calves, respectively (Wilson et al., 2020). In models that sampled dairy herds within a region of Germany, the incorporation of beef semen increased average profit per cow when compared with the exclusive use of conventional dairy semen; herds that used both sexed dairy semen and conventional beef semen in addition to conventional dairy semen had an even greater average profit per cow (Pahmeyer and Britz, 2020).

To maximize profit in Irish dairy herds that breed seasonally, Ruelle et al. (2021) recommended that sexed dairy semen should be used at the beginning of the breeding season until sufficient pregnancies for replacement females were achieved; after such time, all remaining cows should be bred to beef semen. Profits were greatest in herds with better-than-average cow fertility that used the recommended strategy because they generated the most added-value crossbred calves on top of their necessary replacements (Ruelle et al., 2021). More complex mating scenarios have been simulated to determine the economically optimal proportions of conventional, sexed, and beef semen to use in Scandinavian dairy herds. A Swedish model estimated that income per cow was generally greatest in Swedish Holstein and Swedish Red herds when sexed dairy semen was used on 90% of first-calf heifers and all multiparous cows were mated to beef semen (Clasen et al., 2021). A Danish model yielded similar results; as the proportion of beef semen incorporated into the dairy mating programs increased—from 0% to 33%, from 33% to 60%, and from 60% to 70%—the net return per cow increased (Ettema et al., 2017). Net returns were further increased as the cost of raising heifers increased (Ettema et al., 2017). However, Ettema et al. (2017) also reported that using sexed male beef semen to produce male crossbred calves was less profitable than using conventional beef semen in all scenarios *unless* the cost of sexed dairy semen was equivalent to that of conventional dairy semen. In addition, crossbreeding with beef semen reduced profit per cow when compared to breeding with conventional dairy semen if the price of dairy heifers increased by 20%, or more, or if the price of crossbred calves decreased by 50%, or more (Ettema et al., 2017).

While perhaps to a lesser degree, economic models evaluating the use of sexed and beef semen on dairies have also been generated using parameters specific to regions of the United States. A model based on economic values and herd dynamics in the state of Colorado reported that, on an average, combined approaches using sexed and conventional dairy semen resulted in net gains when compared with use of conventional dairy semen alone; however, using sexed dairy semen to generate sufficient replacement heifers and using beef semen on the remaining cows resulted in net losses (McCullock et al., 2013). McCullock et al. (2013) noted that use of beef semen *could* be a profitable strategy if prices of dairy heifer calves were depressed. The models by McCullock et al. (2013) were developed *prior* to the plummet in milk prices in 2014 and the subsequent devaluation of dairy calves (USDA, 2021). In the 2013 Colorado model, dairy heifer calves were being sold for an average of $400 while in September of 2021 the national average price for a dairy heifer calf less than 2 weeks old was between $18 and $31 per hundredweight (McCullock et al., 2013; USDA, 2021). These findings do align with the Danish model that reported beef × dairy to be financially detrimental when dairy heifer prices were elevated (Ettema et al., 2017).

Despite the demonstrated link to heifer economics in prior studies, a recent analysis estimated that dairy herds that only use conventional dairy semen in their breeding programs are the *least profitable* programs (De Vries, 2020). A breeding protocol that combines the use of sexed dairy semen in heifers and young cows, conventional dairy semen in cows with moderate genetic merit, and beef semen in animals of poor genetic merit was deemed most economically optimal (De Vries, 2020). These data further suggest that dairies that genomic test their females benefit most when using the most economically optimal breeding strategy; but, when optimal mating strategies are not utilized, farms that genomically test females experience the greatest economic losses (De Vries, 2020).

The University of Wisconsin has developed a tool, the Premium Beef on Dairy Program, to predict income from calves over semen cost based on the reproductive performance of a dairy herd, market conditions, and distribution of sexed, conventional, and beef matings within a herd (Li and Cabrera, 2019; Li et al., 2020; Cabrera, 2021). The most recent publication using the program determined that that herds with average reproductive performance achieved maximal income from calves over semen cost when sexed dairy semen was used on all first- and second-breeding heifers, first-breeding primiparous cows, and first-breeding second-lactation cows while remaining heifers were bred to conventional dairy semen and remaining cows were mated to beef semen (Cabrera, 2021). Even as market conditions varied, reproductive performance of a herd was the most limiting factor to the profitability of mating strategies in the Premium Beef on Dairy Program (Li and Cabrera, 2019; Cabrera, 2021).

The body of works presented here generally agree that there is recent opportunity for dairy herds to increase profit by using sexed dairy semen on young, genetically superior, females to generate replacement heifers and beef semen on cows of lower genetic merit. However, it is important to consider the circumstances of these economic times to make relative comparisons among future studies. Obviously, economic values and other factors varied among the models presented. Feed, land, and semen expenses can differ among countries. For example, the Irish analysis assumed the cost of beef semen was about one-third of the price of conventional dairy semen (Ruelle, et al., 2021) while analyses in the U.S. assumed conventional beef and dairy semen cost the same (McCullock et al., 2013; De Vries, 2020; Cabrera, 2021). The actual prices US dairies are paying for conventional dairy and beef semen may differ. However, modeling the costs is advantageous because the economic analyses discussed thus far are dependent on the value beef × dairy crossbred calves can achieve for a dairy herd when sold prior to weaning. Little research has been published on how the value of crossbred calves changes if dairies raise them for some period of time through weaning, growing, or finishing. In addition, till date, there are no conclusive analyses determining if the recent increase in beef × dairy calf value—realized at the dairy—is retained throughout the beef supply chain to harvest.

## CURRENT UTILIZATION OF BEEF SEMEN IN THE US DAIRY HERD

Questions of economics aside, beef on dairy matings are increasing in the United States. From 2016 to 2019, 95% of the beef × dairy matings recorded by the Council on Dairy Cattle Breeding in the United States used Angus semen (McWhorter et al., 2020). The next most popular beef × dairy service sires were from the Charolais breed, but these matings made up less than 1% of the total beef × dairy matings (McWhorter et al., 2020). Interestingly, Gelbvieh was the second most popular beef breed to mate to Jerseys and Brown Swiss; Gelbvieh sires were used in 20% of beef × Jersey and 16% of beef × Brown Swiss matings recorded from 2012 to 2019 (McWhorter et al., 2020). From 2010 to 2017, 33% of dairy herds in the Western US reported mating at least a portion of their cows to beef semen (Pereira et al., 2020a). However, by 2020, the majority (77%) of California dairy producers surveyed were incorporating beef semen into their mating protocols (Pereira et al., 2020b). Again, Angus semen was used by most California dairies (65% of producers surveyed), but instead of Charolais in this instance Angus was followed in popularity by Wagyu (used by 12% of producers surveyed) and Limousin (used by 9% of producers surveyed; Pereira et al., 2020b). Reproductive performance, parity, milk production, and genetic merit determined by genomic testing were all cited as reasons to mate a cow to beef semen (Pereira et al., 2020b). Records from the Dairy Herd Information Association from 2010 to 2017 reflect survey results, but they indicate greater adoption of beef semen in herds with poor conception rate and that beef semen was primarily used on cows in their third parity or greater (Pereira et al., 2020a).

## BEEF × DAIRY SELECTION AND MANAGEMENT CONSIDERATIONS

Till date, there has not been an analysis of what drives beef × dairy sire selection in the United States. In some European countries that perform national evaluations on their cattle, specific beef × dairy economic selection indices have been derived to aid producers in bull selection. The beef × dairy selection index in Ireland puts about half of its emphasis on calving and gestation length and half of its emphasis on economically important growth and carcass parameters (Berry et al., 2019). In Scandinavian countries, 2 beef × dairy selection indices have been developed; the first index for calving related traits and the second one for carcass performance (Davis et al., 2019). Genetic parameters are estimated for a variety of beef breeds in Irish and Scandinavian cattle populations, and index values can be weighted for respective beef and dairy breeds used in matings (Berry et al., 2019; Davis et al., 2019). Unfortunately, the European beef × dairy selections indices are unlikely to translate to the U.S. cattle population. European carcasses are valued primarily on lean muscle while carcasses in the United States are priced on a grid of yield and quality with premiums afforded for intramuscular fat (Tatum et al., 2006). Furthermore, the genetic population bases are different between beef and dairy animals in the United States and those in other countries, even when breed names may be similar.

Beef × dairy selection indices for beef sires in the United States have progressed slowly, though private organizations have developed some tools to aid in the selection of beef sires to breed to dairy females. For example, the American Angus Association has developed two separate indices to aid dairy producers in selecting Angus bulls to mate to either Holstein or Jersey dams (Miller et al., 2021). Calving ease is included in the Angus indices along with feed intake and carcass traits economically relevant to US production systems (Miller et al., 2021). In addition, yearling is discounted in the Angus × Holstein index to prevent oversized carcasses at slaughter (Miller et al., 2021). A similar index has been jointly developed by Holstein Association USA and American Simmental Association to recommend SimAngus bulls to mate to Holstein cows (Holstein Association USA, 2021). Of these indices, and other beef bull recommendations published by stud companies, no index weights or derivation methods of economic values are publicly available. Additionally, while European genetic evaluations derive separate expected progeny difference (**EPD**) values for beef bulls based on their beef × dairy progeny, current EPDs for bulls marketed for beef × dairy matings in the United States are based on data from native beef progeny (Berry et al., 2019; Davis et al., 2019; Spangler, 2021). Private progeny testing is being performed on beef × dairy progeny by some stud companies, but EPDs have yet to be published (Johnson et al., 2021). It is unclear if the bulls that dairy producers are being encouraged to select for beef × dairy matings will actually produce crossbred calves that are optimal for the US beef production system.

To achieve desirable phenotypes, that meet the needs of both US dairy and beef producers, intentional selection criteria should be used when beef × dairy matings are made. Simple-trait selection and multiple-trait selection, using selection indices, can help producers achieve beef × dairy breeding goals. Despite this knowledge, selection criteria remain poorly defined for crossbreeding scenarios. To achieve the desirable phenotypic performance of beef × dairy calves from birth to the packer, there are a few important aspects to consider when selecting a beef bull to use for terminal breeding in the dairy herd.

### Calfhood Phenotype

Coat color is one basic phenotype that may add value to preweaned beef × dairy calves. The US beef production system often is willing to pay a premium for black calves because they have the potential to earn additional premiums at slaughter if they have the other attributes to qualify for the Certified Angus Beef program. Thus, most beef × dairy matings have continued to emphasize the black hide which is why Angus sires contribute to the majority of beef × dairy matings in the country (McWhorter et al., 2020; Pereria et al., 2020b). The genetics of cattle coat coloration are well-understood. In most instances, black coat color is dominant to red (Olson, 1999; Dorshorst et al., 2015). In the United States, the Angus breed has had significant influence on the genetic composition of other beef breeds, such as Limousin and Simmental. As such, commercially available semen from many Limousin and Simmental sires are homozygous black. Because black is dominant to red, red beef breeds mated to Holsteins can also have black progeny, but if mated to red or red carrier Holsteins the crossbred calves could be red. The white spots on Holsteins are recessive. Simmental cattle carry the white spotting loci, but most other popular beef breeds do not (Olson, 1999; Reinsch et al., 1999). Beef × Holstein offspring are typically solid colored unless the recessive spotting locus was also inherited from a Simmental sire. The white-face and markings present in the Hereford breed are inherited separately from the piebald spotting expressed by Holsteins and are dominant to solid coloration (Olson, 1999). The white coloration of Herefords is not expressed above the legs or behind the neck, and thus does not disqualify such cattle that are otherwise black from qualifying for Certified Angus Beef premiums. Other beef breeds, like Charolais, carry incompletely dominant genes for dilution of coat color. Charolais cattle heterozygous for dilution appear to have light red or smoke gray coats, while those homozygous for dilution appear white (Olson, 1999). Charolais × dairy calves will never qualify for CAB premiums because Charolais are homozygous for dilution, thus will always produce a calf with diluted coloration (Gutiérrez-Gil et al., 2007).

Polledness is another trait that can be considered beneficial, as dehorning calves creates additional labor for calf growers and is an additional stressor during calfhood. Selection for black coat color and polledness is, again, reflected in surveys of beef × dairy crossbreeding in the United States where Angus semen is most popular (McWhorterer et al., 2020; Pereia et al., 2020b). It is feasible that some beef × dairy calves will be polled regardless of sire genetics, but ≤2% of US Brown Swiss, Holsteins, and Jerseys are naturally hornless; thus, the small proportion of polled genetics that dairy cows contribute to the beef × dairy progeny is likely trivial in comparison to beef sires (Cole et al., 2015). Although certainly relevant, polledness and black hides contribute only a fraction of crossbred calf value. Health, growth, efficiency, and carcass characteristics all contribute to profitable crossbred calves. It is possible that improving those most profitable traits may come with horns or colored hides.

Calf health is vital to the value and success of any preweaned calf marketed. Calf mortality can be selected for; thus, beef × dairy service sire selection can impact calf mortality rate. To that end, calf mortality makes up a small proportion of the index used in Irish beef × dairy programs (Berry et al., 2019). However, in the United States neither beef nor dairy cattle genetic evaluations include calf health or mortality traits. An analysis of Scandinavian beef × dairy calves determined that Belgian Blue-sired calves had the lowest mortality rates (8.2%) while Blonde d’Aquitaine-sired calves had the highest (13%; Davis et al., 2020). Both breeds are double-muscled breeds that are not common in US production systems. The other beef breeds evaluated in the Scandinavian population are more commonly used in beef × dairy matings in the United States. They ranked from least to greatest mortality as: (1) Charolais (9.4%), (2) Simmental (9.5%), and (3) Limousin (12.7%; Davis et al., 2020). Calf respiratory disease, scours, and stayability to 1 year of age are estimated to be poorly heritable in US dairy calves (Haagen et al., 2021). Less heritable traits are often most impacted by heterosis, leading some to suggest that beef × dairy calves could have health advantages over purebred dairy calves (Weaber, 2021). However, there is currently little evidence to confirm such a hypothesis.

Among Holstein heifers and Limousin × dairy heifer and bull calves no variation in respiratory, scours, fecal scores, or general appearance scores was observed (Arens et al., 2021). In one scenario, healthy Angus × Holstein calves fed milk ad libitum over 60 days consumed 20 L more than healthy Holstein calves; but, over the same period, crossbred calves with consolidation in both lungs consumed 37 L fewer of milk than Holstein calves with similar lung consolidation (Steckler et al., 2019). The results suggest that when challenged, Angus × Holstein calves reduce their feed intake more severely than Holstein calves.

Though calf health and mortality can be genetically selected for, there is little evidence that beef × dairy calves have health advantages over purebred dairy calves. As such, good calf management practices need to occur on the dairy to ensure calf health and maintain the value of beef × dairy preweaned calves. Genetic analyses of calf stayability and scours performed by Haagen et al. (2021) suggested that calves may not be able to express genetic health advantages under poor management conditions. Meanwhile, surveys of North American dairy producers indicate that heifer care is often prioritized over the care of male calves that will not enter the milking herd (Creutzinger et al., 2021; Wilson et al., 2021). Of Canadian dairy farmers surveyed, 9% indicated that bull calves did not always receive colostrum, 60% reported that bull calves did not always get their navels dipped in sanitizer, 88% did not vaccinate bull calves, and 83% fed bull calves less than heifer calves (Renaud et al., 2017). Native beef calves that experience failure of passive immunity transfer from poor quality or an inadequate volume of colostrum were over five times more likely to die prior to weaning, over three times more likely to experience morbidity prior to weaning, and over three times more likely to experience health events on the feedlot (Wittum and Perino, 1995). Perceived cost of calf care related to calf value was cited as one reason bull calves receive suboptimal care (Wilson et al., 2021); however, analysis of Canadian auction data revealed that male calves with depressed attitude were valued about $45 CAD less than calves that appeared healthy and alert (Wilson et al., 2020). While it is unclear if the added value of beef × dairy calves may incentivize better care than purebred dairy bull calves historically received, it is clear that sick calves will be devalued.

Crossbred calf size may be one additional phenotypic advantage to add value to preweaned beef × dairy calves because calves are typically sold by weight at auctions. Even when not auctioned by weight, each additional kg of weight increased the value of Canadian male dairy calves sold by $6 (Wilson et al., 2020). At birth, Limousin × dairy bull calves were 6 kg heavier than Holstein heifer calves while Limousin × dairy heifer calves did not differ in birth weight from Holstein heifers (Arens et al., 2021). Charolais × Friesian and Simmental × Friesian calves were 5 kg and 3 kg heavier at birth than purebred Friesian calves, respectively, whereas Hereford and Angus calves were 1 kg and 3 kg lighter, respectively (Everitt et al., 1978). Charolais × Brown Swiss calves were 18%–28% heavier than Angus and Hereford-sired calves out of Brown Swiss dams (Pahnish et al., 1969). While heavier birth weights have been correlated with increased calving difficulties, there is a clear variation both within and among beef sire breed for birth weight of beef × dairy calves (Everitt et al., 1978). Thus, it may be most valuable to select for moderate birth weights of beef × dairy calves to mitigate the risk of calving problems while still generating a calf that is large enough to market.

### Growth Performance

When comparing beef service sire breeds mated to dairy animals throughout the literature, growth performance data are variable. Caution should be used when applying the available data to beef on dairy decisions in the United States. Many of the available publications on the growth and efficiency of beef × dairy animals are now decades old and significant genetic progress has been made in many of the beef breeds discussed. At the same time, dairy breeds have been selected to produce milk more efficiently, which may result in purebred dairy steers having even more notable differences in growth and efficiency than their crossbred peers. Furthermore, many of the published data were collected internationally. Not only do international genetic lineages differ substantially from those in US animals, but also nutrition and management strategies vary based on climate and feed availability. To account for nutritional differences, the following sections encompassing growth performance and carcass characteristics will separate data based on nutritional management. Data from cattle grown and finished on diets that contained at least 50% forage on a dry matter (**DM**) basis will be referred to as “Forage Finished” systems and data from cattle intensively finished on diets that contained greater than 50% concentrates (DM basis) will be referred to as “Concentrate Finished” systems. The data discussed in this section is summarized in Table 1.

**Table 1.** Growth performance characteristics of beef × dairy animals across the literature. In some instances, purebred dairy and beef animal data is included for comparison. Means separations and *p*-values are not reported here, as any significant differences are discussed in the text of the review.

Feeding system	Reference	Sire breed	Dam breed	Sex	Slaughter criteria	Age at slaughter, days	Days on feed	ADG, kg/day	DMI, kg/day	FE, kg gain/kg feed
Forage finished	Southgate et al. (1982)	Angus	British Friesian	Steer	16 mo. old	438	—	0.84	4.6	0.203
		Charolais	British Friesian			523	—	0.94	5.3	0.192
		Devon	British Friesian			440	—	0.86	4.6	0.213
		Hereford	British Friesian			453	—	0.85	4.4	0.217
		Simmental	British Friesian			506	—	0.93	5.2	0.201
		South Devon	British Friesian			491	—	0.9	5.1	0.199
		Sussex	British Friesian			460	—	0.86	4.7	0.208
		British Friesian	British Friesian			500	—	0.85	4.9	0.192
		Angus	British Friesian		24 mo. old	685	—	0.63	4.6	0.151
		Charolais	British Friesian			743	—	0.79	5.5	0.16
		Devon	British Friesian			705	—	0.67	4.8	0.158
		Hereford	British Friesian			698	—	0.68	4.6	0.165
		Simmental	British Friesian			744	—	0.73	5.4	0.149
		South Devon	British Friesian			733	—	0.72	5.2	0.155
		British Friesian	British Friesian			738	—	0.7	5.4	0.148
	Southgate et al. (1988)	Charolais	British Friesian	Steer	16 mo. old	526	—	0.92	4.75	0.196
		Hereford	British Friesian			450	—	0.83	4.18	0.198
		Limousin	British Friesian			516	—	0.83	4.26	0.2
		Lincoln Red	British Friesian			447	—	0.87	4.36	0.2
		Simmental	British Friesian			527	—	0.88	4.51	0.198
		South Devon	British Friesian			494	—	0.81	4.34	0.191
		Sussex	British Friesian			448	—	0.87	4.31	0.205
		British Friesian	British Friesian			523	—	0.83	4.75	0.175
		Canadian Holstein	Canadian Holstein			589	—	0.82	5.04	0.164
		Charolais	British Friesian		24 mo. old	772	—	0.75	4.66	0.165
		Hereford	British Friesian			714	—	0.66	4.21	0.161
		Limousin	British Friesian			760	—	0.72	4.37	0.17
		Lincoln Red	British Friesian			717	—	0.67	4.26	0.161
		Simmental	British Friesian			744	—	0.72	4.53	0.163
		South Devon	British Friesian			715	—	0.73	4.4	0.171
		Sussex	British Friesian			735	—	0.69	4.21	0.167
		British Friesian	British Friesian			768	—	0.67	4.65	0.149
		Canadian Holstein	Canadian Holstein			814	—	0.7	4.91	0.146
	Davies et al. (1999)	Charolais	Holstein-Friesian	Steer	4L fatness	—	233	1.09	—	—
		Piedmontese	Holstein-Friesian			—	264	0.98	—	—
		Charolais	Holstein-Friesian	Heifer	440 kg	—	256	0.87	—	—
		Piedmontese	Holstein-Friesian			—	287	0.82	—	—
	Muir et al. 2000)	Hereford	Friesian	Steer	7 mm of fat at 12th rib by ultrasound	756	—	—	—	—
		Friesian	Friesian			812	—	—	—	—
		Hereford	Hereford			980	—	—	—	—
	Huuskonen et al. (2013a)	Angus	Holstein	Heifer	—	471	—	—	—	—
		Blonde d’Aquitanie	Holstein		—	469	—	—	—	—
		Charolais	Holstein		—	464	—	—	—	—
		Hereford	Holstein		—	478	—	—	—	—
		Limousin	Holstein		—	476	—	—	—	—
		Simmental	Holstein		—	473	—	—	—	—
		Holstein	Holstein		—	486	—	—	—	—
		Angus	Norwegian Red		—	478	—	—	—	—
		Blonde d’Aquitanie	Norwegian Red		—	471	—	—	—	—
		Charolais	Norwegian Red		—	470	—	—	—	—
		Hereford	Norwegian Red		—	477	—	—	—	—
		Limousin	Norwegian Red		—	477	—	—	—	—
		Simmental	Norwegian Red		—	481	—	—	—	—
		Norwegian Red	Norwegian Red		—	492	—	—	—	—
	Huuskonen et al. (2013b)	Angus	Holstein	Bull	—	592	—	—	—	—
		Blonde d’Aquitanie	Holstein		—	582	—	—	—	—
		Charolais	Holstein		—	575	—	—	—	—
		Hereford	Holstein		—	592	—	—	—	—
		Limousin	Holstein		—	586	—	—	—	—
		Simmental	Holstein		—	582	—	—	—	—
		Holstein	Holstein		—	587	—	—	—	—
Concentrate finished	Urick et al. (1974)	Angus	Brown Swiss	Steer	465 kg	437	—	—	—	—
		Charolais	Brown Swiss			406	—	—	—	—
		Hereford	Brown Swiss			418	—	—	—	—
	Fahmy and Lalande (1975)	Charolais	Holstein	Steer	454 kg	164	—	1.13	4.78	0.236
		Hereford	Holstein		454 kg	199	—	0.93	4.52	0.206
		Charolais	Holstein		544 kg	283	—	0.98	4.78	0.205
		Hereford	Holstein		544 kg	316	—	0.88	4.47	0.197
		Charolais	Holstein		635 kg	410	—	0.9	4.67	0.193
		Hereford	Holstein		635 kg	442	—	0.83	4.62	0.179
	Bech Andersen et al. (1977)^1^	Blonde d’Aquitaine	Danish Red or Black Pied Danish	Bull	300 kg, 12 mo. old, or 15 mo. old	—	—	1.265	—	3.981
		Charolais	Danish Red or Black Pied Danish			—	—	1.267	—	3.971
		Chianina	Danish Red or Black Pied Danish			—	—	1.225	—	4.121
		Danish Red and White	Danish Red or Black Pied Danish			—	—	1.229	—	4.081
		Hereford	Danish Red or Black Pied Danish			—	—	1.173	—	4.171
		Limousin	Danish Red or Black Pied Danish			—	—	1.184	—	4.231
		Romagnola	Danish Red or Black Pied Danish			—	—	1.237	—	4.091
	Forrest (1977)	Charolais	Holstein	Steer	500 kg	—	—	0.99 to 1.69	—	—
		Charolais	Holstein	Heifer		—	—	0.98 to 1.25	—	—
		Holstein	Holstein	Steer		—	—	0.95 to 1.47	—	—
	Forrest (1980)^2^	Simmental	Holstein	Steer	500 kg	—	—	1.02 to 1.60	—	4.37 to 5.23^2^
		Simmental	Holstein	Heifer		—	—	1.00 to 1.09	—	5.51 to 6.38^2^
		Holstein	Holstein	Steer		—	—	1.01 to 1.61	—	4.61 to 5.55^2^
	Forrest (1981)^2^	Limousin	Holstein	Steer	500 kg	—	—	1.03 to 1.53	5.97 to 9.90	3.62 to 6.54^2^
		Limousin	Holstein	Heifer		—	—	0.93 to 1.06	5.60 to 10.02	3.36 to 4.82^2^
		Holstein	Holstein	Steer		—	—	1.01 to 1.46	7.03 to 11.62	3.84 to 5.83^2^
	Jaborek et al. (2019a)	Angus	Jersey	Steer	523 kg	533	304	1.05	7.72	0.133
		Red Wagyu	Jersey			562	331	0.97	6.8	0.142
		SimAngus	Jersey			539	314	1.08	7.35	0.135
		Jersey	Jersey			602	346	0.85	6.67	0.123
	Jaborek et al. (2019b)	Angus	Jersey	Steers and Heifers	523 kg (steer) and 500 kg (heifer)	537	311	1.01	7.37	0.137
		Red Wagyu	Jersey			573	345	0.89	6.47	0.133
		SimAngus	Jersey			552	322	1.01	7.08	0.132
	Rezagholivand et al. (2021)	Angus	Holstein	Steers and Heifers	11 mo. on feed	—	—	1.34	8.81	0.177
		Charolais	Holstein			—	—	1.38	7.79	0.152
		INRA 95	Holstein			—	—	1.34	8.83	0.161
		Limousin	Holstein			—	—	1.35	8.39	0.156
		Holstein	Holstein			—	—	1.25	8.02	0.177

Feed efficiency is reported as Scandinavian feed units per kg of gain.

Feed efficiency is reported as total digestible nutrients per kg of gain.

One reported advantage of beef × dairy crossbreds is increased average daily gain (**ADG**) compared to purebred dairy counterparts. In addition to being able to rapidly gain weight, it is economically advantageous for beef × dairy animals to efficiently convert feed to beef. Beef sire breed may impact the ADG, dry matter intake (**DMI**), and subsequent feed efficiency of beef × dairy calves. Even prior to weaning, the ADG of Charolais × Brown Swiss steers was 7% greater than Angus × Brown Swiss steers (Pahnish et al., 1969). Charolais × Brown Swiss heifers and steers were 9% and 10% heavier at weaning than Angus × Brown Swiss calves of respective sexes (Pahnish et al., 1969). Likewise, Charolais × Brown Swiss heifers weaned 8% heavier than Hereford × Brown Swiss heifers (Pahnish et al., 1969). Conversely, no differences were detected in preweaning ADG between Holstein and Limousin × dairy calves, but Limousin × dairy heifer and bull calves consumed less milk, fed *ad libitum*, and were, therefore, more feed efficient (Arens et al., 2021). These data are relevant only if calves are sold from the production system at weaning. Thus, differences in ADG, DMI, and feed efficiency between dairy and beef × dairy cattle in both forage-based and concentrate-based (or grain-based) feeding systems will be explored. While discussing both bulls (or steers) and heifers, it is important to remember that beef × dairy bull calves are typically valued at a premium over beef × dairy heifer calves at sale. Meanwhile, crossbred calves of both sexes are replacing Holstein steers in the production system; thus, it is valuable to compare both sexes of crossbreds to Holstein steers.

#### Forage Finished.

 Among beef × British Friesian steers slaughtered at 16 and 24 months of age and sired by 8 beef sire breeds, the ADG of Charolais-sired steers was 1%–25% greater than the ADG of steers with other beef sire breeds (Southgate et al., 1982; 1988). Charolais × British Friesian steers also gained 0.05–0.10 kg more weight per day than purebred British Friesian and Canadian Holstein steers and converted digestible organic matter to gain 14%–17% more efficiently than the purebred dairy steers (Southgate et al., 1982, 1988). The authors distinguished between British Friesian and Canadian Holsteins because, at the time, the US dairy population was mostly Holstein–Friesian though Holstein genetics, like the Canadian Holstein genetics referenced in the study, are now the standard. In some scenarios, Hereford, Limousin, and Simmental-sired steers had greater ADG or converted feed to weight gain more efficiently than British Friesian and Canadian Holstein steers, but not with the same consistency nor at the same magnitude as the Charolais × British Friesian steers (Southgate et al., 1982, 1988). While Charolais-sired steers were 42–63 days younger than Holstein steers at slaughter, Charolais × Friesian steers were slaughtered at a similar age as Friesian steers and were 32–85 days *older* at slaughter than steers sired by British beef breeds (Southgate et al., 1982, 1988). Finnish data reported that average age at slaughter was more similar between dairy breeds and different beef × dairy crosses (Huuskonen et al., 2013a, 2013b) than previously reported (Southgate et al., 1982, 1988). In these observations, the greatest differences were between Charolais × Holstein and Norwegian Red heifers, with the Charolais crosses 28 days younger at slaughter, and between Charolais × Holstein bulls and Angus × Holstein bulls, with the Charolais crosses 17 days younger at slaughter (Huuskonen et al., 2013a, 2013b). In a separate study, Charolais × Holstein-Friesian heifers and steers were on feed 31 days less than Piedmontese × Holstein–Friesian heifers and steers when fed to target end body weights because Charolais × Holstein–Friesian steers and heifers had 11% and 6% greater ADG greater than Piedmontese × Holstein–Friesian steers and heifers, respectively (Davies et al., 1999). When finished exclusively on grass, purebred Hereford steers reached a desired level of fattening within 27 months; however, it took Hereford × Friesian steers 29 months to achieve the same level of fattening and it took purebred Friesians 35 months (Muir et al., 2000).

Thus, when finished on forage-based diets, beef × dairy cattle often gained more weight, spent fewer days on feed, and were more efficient than dairy cattle. Charolais-sired beef × dairy cattle were typically more efficient in forage-based systems than the crosses sired by British beef breed sires; however, in some scenarios the Charolais-sired calves were older at slaughter, which would increase yardage and feed costs. Scandinavian genetic evaluations support that Continental beef breeds have advantages in gain when used in beef × dairy matings.

#### Concentrate Finished.

 Beef × dairy cattle have demonstrated similar advantages in ADG over dairy steers when finished in concentrate-based feeding systems that are common in the United States. Although no means separation tests were performed, a comparison of 7 sire breeds reported that Charolais × dairy bull calves had the greatest ADG (1.27 kg/day) while Hereford-sired bull calves had the least (1.17 kg/day; Bech Andersen et al., 1977). Subsequently, Charolais-sired bull calves were the most efficient, requiring 7% less feed per kg of gain than the least efficient Limousin-sired bull calves (Bech Andersen et al., 1977).

Just as in the forage-based systems, Continental sires, specifically Charolais sires, often seem to be at an advantage in concentrate-based beef × dairy systems. When finished to a target weight, Angus × Brown Swiss steers were 31 days older at slaughter than Charolais × Brown Swiss steers (Urick et al., 1974). Fahmy and Lalande (1975) observed the ADG of Canadian Charolais × Holstein steers was, on average, 14% greater than that of Hereford × Holstein steers when fed to three target end weights. The Charolais × Holsteins also took 32–35 fewer days to achieve target end weights and were 8% more efficient at converting feed to gain than Hereford × Holsteins (Fahmy and Lalande, 1975). The ADG of Angus, Charolais, Limousin, and INRA 95-sired steers out of Holstein dams were 7%–10% greater than those of purebred Holstein steers fed over an 11-month period (Rezagholivand et al., 2021). The INRA 95 is a French composite breed developed from Charolais, Blonde d’Aquitaine, Limousin, Maine-Anjou, Piedmontese, and Belgian Blue genetics. While uncommon in the United States, INRA 95 sires are popular in beef × dairy matings internationally. The DMI of Charolais × Holstein calves was 0.23–1.04 kg less per day than that of the other breeds, which made them 9%–13% more feed efficient than calves sired by Angus, INRA 95, or Limousin bulls (Rezagholivand et al., 2021).

A series of studies (Forrest, 1977, 1980, 1981) conducted evaluating beef × Holstein heifers and steers sired by Continental breeds in comparison to purebred Holstein steers yielded contradictory results to the aforementioned data. Limousin × Holstein steers and Holstein steers gained 0.08–0.47 kg more, daily, than Limousin × Holstein heifers (Forrest, 1981). However, both Limousin × Holstein heifers and steers consumed 16%–18% less feed per day and were therefore 18%–21% more feed efficient than purebred Holstein steers (Forrest, 1981). The ADG of Charolais × Holstein steers and heifers did not differ significantly from that of purebred Holstein steers (Forrest, 1977). Purebred Holstein steers and Simmental × Holstein steers gained 12%–50% more weight per day than heifers of the same cross (Forrest, 1980). It should be noted that management factors may have influenced some of the sex differences observed as Holstein and crossbred steers received hormonal implants while crossbred heifers did not (Forrest, 1977, 1980, 1981). Sex differences in ADG were not surprising as heifers often finish lighter than steers. However, the lack of differences in ADG between beef × Holstein steers and Holstein steers contradict other studies comparing intensively fed beef × dairy steers to dairy steers. This may be due to the age of the studies presented.

Lucas et al. (2021) evaluated historic 205-day weaning weight and yearling weights of animals in the United States with dairy and Simmental genetic influence. Every percentage point increase of Simmental genetic influence resulted in increased weaning weight by 0.29 kg and yearling weight by 0.44 kg. Though the work of Lucas et al. (2021) did not directly evaluate ADG, it suggested that breed composition influences animal growth to weaning and yearling weight.

While Holsteins are by far the most popular dairy breed in the United States, it is crucial to note that preweaned Jersey bull calves have been undervalued longer than Holstein bull calves. To that end, recent work funded by the American Jersey Cattle Association suggests that Angus genetics may be most beneficial in beef × Jersey systems. Angus, SimAngus, and Red Wagyu-sired steers out of Jersey dams gained 0.12–0.23 kg more, daily, than purebred Jersey steers (Jaborek et al., 2019a). These differences in ADG may, in part, reflect that Jersey steers consumed between 0.13 and 1.05 kg less DM per day than their crossbred counterparts (Jaborek et al., 2019a). As such, Jersey steers were on feed 15 days longer than Red Wagyu × Jersey steers, 32 days longer than SimAngus × Jersey steers, and 42 days longer than Angus × Jersey steers (Jaborek et al., 2019a). Though Red Wagyu-sired steers were on feed longer than Angus-sired calves, Red Wagyu-sired steers were more feed efficient than the other breeds in part due to their reduced feed intake (Jaborek et al., 2019a). A similar study reported that, on average, Angus × Jersey and SimAngus × Jersey heifers and steers gained 0.12 kg per day more than Red Wagyu × Jersey heifers and steers (Jaborek et al., 2019b). Angus × Jersey calves consumed 14% more DM per day and spent 34 fewer days on feed than Red Wagyu-sired calves while SimAngus-sired calves consumed 10% more feed and spent 24 fewer days on feed than Red Wagyu × Holsteins (Jaborek et al., 2019b). However, feed conversion rates ultimately did not differ between sire breeds (Jaborek et al., 2019b). Over the past several decades, British breeds in the United States have been selected for heavier body weights which has resulted in Angus exceeding continental breeds in direct breed effect for mature body weight (Zimmermann et al., 2021). These trends may impact comparisons between the historic and the more recent beef × dairy data.

One of the greatest challenges to the success of beef × dairy systems is communication across industries. Beef × dairy calves are yielding profit to the dairy farm, but that profit potential is not always realized throughout the supply chain, in part, due to the variation in growth performance discussed above. Till date, EPDs for ADG and DMI are only estimated by Angus, Red Angus, and Hereford breed associations in the United States (American Angus Association, 2021; American Hereford Association, 2021; Red Angus Association of America, 2021). Because relatively few breed associations generate EPDs for ADG and DMI, there are no across-breed EPD conversion factors for the traits. Thus, weaning weight and yearling weight EPDs are used to gauge progeny growth because they can be compared across breeds. Using the available EPDs for growth traits should give dairy producers the ability to make informed selection decisions regarding growth when selecting from a variety of available beef breeds. In addition, selecting on these criteria may make the resulting calves more desirable through the entire beef supply chain.

### Carcass Characteristics

United States beef production systems value meat quality more than international beef production systems. Thus, carcass value ultimately determines the net value of beef × dairy cattle because most fed beef cattle in the United States are sold on a carcass grid system. The carcass characteristics discussed in this review are predominantly those that hold value on the grid in the United States, with other metrics discussed where relevant.

The “grid” in the United States is based on the USDA Yield Grade and USDA Quality Grade. Carcass weight, backfat thickness, and ribeye area are primary drivers of USDA Yield Grade and, in cattle under 30 months of age, marbling score is the sole driver of USDA Quality Grade. Carcass weight and dressing percentage are discussed because grid prices are based on each hundredweight (45 kg) of a dressed carcass. Base grid price is set for carcasses that achieve a Yield Grade of 3 and Quality Grade of Choice; premiums are added to carcasses with Yield Grades 1 and 2 and Prime Quality Grades while discounts are applied to carcasses with Yield Grades 4 and 5 and Select Quality Grades. When breeding beef × dairy, breeding goals should focus on creating cattle that avoid the discounts that purebred Holsteins have historically suffered, such as poor dressing percentages, in grid-based systems. Data presented throughout this section is summarized in Table 2. For each relevant carcass trait, data will again be separated by finishing diet.

**Table 2.** Carcass characteristics of beef × dairy animals across the literature. In some instances, purebred dairy and beef animal data is included for comparison. Means separations and *p*-values are not reported here, as any significant differences are discussed in the text of the review.

Feeding system	Reference	Sire breed	Dam breed	Sex	Slaughter criteria	Carcass weight, kg	Dressing %	Ribeye area, cm^2^	Backfat, mm	Marbling score	Shear force, kg force
Forage finished	Fahmy and Lalande (1975)	Charolais	Holstein	Steer	454 kg	—	55.6	67.0	3.7	—	—
		Hereford	Holstein			—	55.0	63.0	5.4	—	—
		Charolais	Holstein		544 kg	—	56.1	69.1	5.0	—	—
		Hereford	Holstein			—	56.9	66.6	7.6	—	—
		Charolais	Holstein		635 kg	—	57.0	80.5	4.4	—	—
		Hereford	Holstein			—	56.5	66.5	12.6	—	—
	Kempster et al. (1982)	Angus	British Friesian	Steer	16 mo. old	179	48.5	52.0	—	—	—
		Charolais	British Friesian			261	51.5	69.0	—	—	—
		Devon	British Friesian			186	48.5	52.0	—	—	—
		Hereford	British Friesian			195	49.1	51.0	—	—	—
		Simmental	British Friesian			244	50.4	65.0	—	—	—
		South Devon	British Friesian			227	50.1	59.0	—	—	—
		Sussex	British Friesian			201	49.7	58.0	—	—	—
		British Friesian	British Friesian			216	49.6	56.0	—	—	—
		Angus	British Friesian		24 mo. old	221	48.7	44.0	—	—	—
		Charolais	British Friesian			317	51.6	64.0	—	—	—
		Devon	British Friesian			242	49.0	49.0	—	—	—
		Hereford	British Friesian			245	50.1	49.0	—	—	—
		Simmental	British Friesian			286	50.4	58.0	—	—	—
		South Devon	British Friesian			279	50.7	56.0	—	—	—
		British Friesian	British Friesian			266	49.2	53.0	—	—	—
	Kempster et al. (1988)	Charolais	British Friesian	Steer	16 mo. old	277	53.2	69.8	—	—	—
		Hereford	British Friesian			203	50.9	54.7	—	—	—
		Limousin	British Friesian			247	53.7	66.9	—	—	—
		Lincoln Red	British Friesian			213	50.4	57.6	—	—	—
		Simmental	British Friesian			257	52.2	66.9	—	—	—
		South Devon	British Friesian			231	52.3	60.4	—	—	—
		Sussex	British Friesian			210	51.1	60.2	—	—	—
		British Friesian	British Friesian			240	51.3	60.1	—	—	—
		Canadian Holstein	Canadian Holstein			265	50.5	58.4	—	—	—
		Charolais	British Friesian		24 mo. old	332	53.0	71.7	—	—	—
		Hereford	British Friesian			261	50.6	59.7	—	—	—
		Limousin	British Friesian			312	53.0	73.6	—	—	—
		Lincoln Red	British Friesian			259	50.3	61.4	—	—	—
		Simmental	British Friesian			299	51.2	69.1	—	—	—
		South Devon	British Friesian			287	51.2	68.2	—	—	—
		Sussex	British Friesian			279	51.5	68.0	—	—	—
		British Friesian	British Friesian			282	50.3	62.2	—	—	—
		Canadian Holstein	Canadian Holstein			311	50.1	60.7	—	—	—
	Davies et al., 1999 ^1^	Charolais	Holstein-Friesian	Steer	4L fatness	264	56.9	—	3.2^1^	—	—
		Piedmontese	Holstein-Friesian			274	58.3	—	2.7^1^	—	—
		Charolais	Holstein-Friesian	Heifer	440 kg	243	55.8	—	2.9^1^	—	—
		Piedmontese	Holstein-Friesian			259	57.8	—	2.7^1^	—	—
	Muir et al. (2000)	Hereford	Friesian	Steer	7mm at 12th rib by ultrasound	329	54.3	—	7.9	—	6.63
		Friesian	Friesian			367	51.3	—	7.9	—	7.34
		Hereford	Hereford			318	53.3	—	9.2	—	5.89
	Huuskonen et al. (2013a) ^1^	Angus	Holstein	Heifer		221	—	—	3.3^1^	—	—
		Blonde d’Aquitanie	Holstein			238	—	—	2.5^1^	—	—
		Charolais	Holstein			246	—	—	3.0^1^	—	—
		Hereford	Holstein			231	—	—	3.7^1^	—	—
		Limousin	Holstein			237	—	—	3.0^1^	—	—
		Simmental	Holstein			235	—	—	3.1^1^	—	—
		Holstein	Holstein			208	—	—	2.7^1^	—	—
		Angus	Norwegian Red			226	—	—	3.6^1^	—	—
		Blonde d’Aquitanie	Norwegian Red			234	—	—	2.5^1^	—	—
		Charolais	Norwegian Red			242	—	—	2.9	—	—
		Hereford	Norwegian Red			232	—	—	3.8	—	—
		Limousin	Norwegian Red			233	—	—	2.9	—	—
		Simmental	Norwegian Red			237	—	—	3.1	—	—
		Norwegian Red	Norwegian Red			292	—	—	2.7	—	—
	Huuskonen et al., 2013b ^1^	Angus	Holstein	Bull		357	—	—	3.0^1^	—	—
		Blonde d’Aquitanie	Holstein			379	—	—	2.1^1^	—	—
		Charolais	Holstein			387	—	—	2.5^1^	—	—
		Hereford	Holstein			366	—	—	3.2^1^	—	—
		Limousin	Holstein			372	—	—	2.5^1^	—	—
		Simmental	Holstein			383	—	—	2.7^1^	—	—
		Holstein	Holstein			333	—	—	2.4^1^	—	—
Concentrate finished	Urick et al. (1974)	Angus	Brown Swiss	Steer	465 kg	274	—	76.6	14.1	14.9	7.28
		Charolais	Brown Swiss			278	—	79.3	9.9	17.1	7.45
		Hereford	Brown Swiss			272	—	75.0	14.3	16.7	8.07
	Bech Andersen et al. (1977)	Blonde d’Aquitaine	Danish Red or Black Pied Danish	Bull	300 kg, 12 mo. old, or 15 mo. old	263	56.2	41.6	—	—	—
		Charolais	Danish Red or Black Pied Danish			255	54.8	37.2	—	—	—
		Chianina	Danish Red or Black Pied Danish			249	54.9	37.1	—	—	—
		Danish Red and White	Danish Red or Black Pied Danish			248	54.1	34.2	—	—	—
		Hereford	Danish Red or Black Pied Danish			236	54.0	32.6	—	—	—
		Limousin	Danish Red or Black Pied Danish			249	56.0	40.1	—	—	—
		Romagnola	Danish Red or Black Pied Danish			457	54.4	66.7	—	—	—
	Forrest (1977)	Charolais	Holstein	Steer	500 kg	277	58.8	78.8	9.4	—	—
		Charolais	Holstein	Heifer		269	57.2	76.9	12.4	—	—
		Holstein	Holstein	Steer		268	56.7	69.2	11.6	—	—
	Forrest (1980)	Simmental	Holstein	Steer	500 kg	272	57.5	74.7	9.6	—	—
		Simmental	Holstein	Heifer		272	57.3	74.5	14.0	—	—
		Holstein	Holstein	Steer		267	56.3	67.2	9.4	—	—
	Forrest (1981)	Limousin	Holstein	Steer	500 kg	283	58.6	76.5	12.9	—	—
		Limousin	Holstein	Heifer		280	59.4	77.9	17.0	—	—
		Holstein	Holstein	Steer		262	55.7	60.2	10.2	—	—
	Baker et al. (1984) ^2^	Angus^2^	Holstein^2^	Bull	12, 15, 18, or 24 mo. old	309	58.9	83.7	3.8	7.6	—
		Brahman^2^	Holstein^2^			325	59.1	84.2	3.6	6.6	—
		Hereford^2^	Holstein^2^			308	55.6	81.7	4.3	7.3	—
		Angus^2^	Jersey^2^			259	57.8	73.5	4.1	8.3	—
		Brahman^2^	Jersey^2^			308	67.5	77.6	4.2	7.7	—
		Hereford^2^	Jersey^2^			249	55.1	75.2	4	8.9	—
		Holstein	Holstein			309	54.0	78.3	3.4	5.3	—
		Jersey	Jersey			209	55.6	61	3.5	5.7	—
	Jaborek et al. (2019a)	Angus	Jersey	Steer	523 kg	334	64.2	73.6	13.7	745	2.76
		Red Wagyu	Jersey			317	63.2	76.4	9.7	687	2.39
		SimAngus	Jersey			332	63.9	73.6	10.4	651	2.48
		Jersey	Jersey			304	61.2	70.3	8.6	586	2.71
	Jaborek et al. (2019b)	Angus	Jersey	Steers and Heifers	523 kg (steer) and 500 kg (heifer)	327	64.1	76.4	14.7	800	2.69
		Red Wagyu	Jersey			306	63.1	74.1	11.1	690	2.44
		SimAngus	Jersey			324	63.3	75.2	11.7	677	2.45
	Rezagholivand et al. (2021)	Angus	Holstein	Steers and Heifers	11 months on feed	280	51	110.1	6.4	—	—
		Charolais	Holstein			307	56	118.5	4.3	—	—
		INRA 95	Holstein			303	55.3	118.7	5.2	—	—
		Limousin	Holstein			298	55.4	104.2	5.6	—	—
		Holstein	Holstein			274	49.9	99.9	5.4	—	—

EUROP fat score is reported to quantify fat cover rather than backfat thickness.

Crossbred progeny could have either breed as sire or dam as they resulted from a diallel experiment. For example, F1 Angus-Holstein bulls could have an Angus dam and a Holstein sire or a Holstein dam and an Angus sire.

### Carcass Weight and Yield

Carcass weight and dressing percentage act as approximate measures of beef yield following the removal of the head, hide, and organs of an animal. Thus, heavier carcasses generally result in greater net profitability in the United States.

#### Forage Finished.

 The average carcass weight of purebred British Friesian steers slaughtered at 16 months of age was 216 kg, which did not differ significantly from Hereford, South Devon, or Sussex-sired steers out of British Friesian dams (Kempster et al., 1982). However, Charolais and Simmental-sired steers had carcasses that were 45 kg and 28 kg heavier, respectively, than purebred British Friesian steers, while carcasses from Angus-sired steers were 37 kg lighter than those from purebred British Friesian (Kempster et al., 1982). Despite these differences, Angus, Charolais, Simmental, and South Devon-sired calves out of British Friesian dams produced 2% more salable meat than British Friesian steers in proportion to their carcass weight (Kempster et al., 1982). When slaughtered at 24 months, the average carcass weight of purebred British Friesian steers was 266 kg and did not differ from the carcass weights of Simmental and South Devon-sired steers (Kempster et al., 1982). However, Charolais × British Friesian steers had carcasses 51 kg heavier while Angus × Friesian steers and Hereford × Friesian steers had carcasses 45 kg and 24 kg lighter, respectively, than purebred British Friesian steers (Kempster et al., 1982). Similar to their younger counterparts, Charolais and Angus-sired steers killed at 24 months of age produced more salable red meat per kg of carcass weight when compared to British Friesian steers of the same age (Kempster et al., 1982). In a later trial, the carcass weights of progeny sired by Continental beef sires (Simmental, Charolais, and Limousin) were not different than purebred dairy steers while carcasses from the British breed (Angus and Hereford) sires remained lighter than the Holstein and Friesian counterparts (Kempster et al., 1988). Again, Continental-sired cattle yielded more saleable red meat per kg of carcass weight than the Holstein and Friesian counterparts (Kempster et al., 1988). Notably, the carcasses of British Friesian steers yielded 3%–4% more meat than Canadian Holsteins, characterizing some of the differences between two dairy breeds that are often considered interchangeable in the United States because Friesian genetics have essentially been bred out of the Holstein population (Kempster et al., 1988). In addition, these data suggest that in the 1980s, despite the differences in final carcass weight, beef × dairy progeny generally yielded more saleable red meat yield than their purebred dairy counterparts. While not all studies report saleable red meat yield, making comparisons across studies challenging, this metric is the truest measure of meat produced from an animal and, as such, is worth discussing when reported.

When compared with the carcasses of purebred Norwegian Red or Holstein heifers, which weighed 202 kg and 208 kg, respectively, heifers sired by Angus, Blonde d’Aquitanie, Charolais, Hereford, Limousin, or Simmental bulls yielded heavier carcasses (Huuskonen et al., 2013a). In fact, the heaviest carcasses were from Charolais-sired heifers had out of Norwegian Red and Holstein dams, at 242 kg and 246 kg, respectively (Huuskonen et al., 2013a). The same was true when comparing the carcasses of Holstein bulls (333 kg) to beef × Holstein crossbred bulls of the aforementioned breeds; Charolais × Holstein bulls had the heaviest carcasses (387 kg; Huuskonen et al., 2013b). The carcasses of Piedmontese × Holstein-Friesian heifers and steers finished on grass were 16 kg and 10 kg heavier, respectively, than carcasses of Charolais × Holstein-Friesian heifers and steers (Davies et al., 1999). In addition, the corresponding dressing percentages of Piedmontese-sired heifers and steers, 57.8% and 58.3%, were also 2.0% and 1.4% greater than those of Charolais-sired heifers and steers (Davies et al., 1999). The Piedmontese breed is typically double muscled, while only some of the Charolais population is. Authors did not discuss whether or not the Charolais sires in these studies had the double muscling mutation. Use of double-muscled breeds is popular in international beef × dairy mating schemes to increase lean tissue yield because fat is not valued in these systems as it is in US beef production. Most US production systems finish cattle in concentrate-based systems to increase the fat deposition in the carcass.

#### Concentrate Finished.

 Carcass weight of grain-fed Charolais × Holstein steers did not differ from purebred Holstein steers but Charolais × Holstein steers had a dressing percentage of 58.8% while the dressing percentage of Holstein steers was 56.7% (Forrest, 1977). Both Simmental × Holstein heifers and steers exceeded purebred Holstein steers in carcass weight by 5 kg and dressed out 1 percentage unit greater than Holstein steers (Forrest, 1980). A similar trend in Limousin × Holstein heifers and steers was observed. The carcass weights of crossbred steers and heifers exceeded those of Holsteins steers by 20 and 18 kg, respectively, while the dressing percentages reflected the difference in weight of Limousin × Holstein steers (58.6%) and heifers (59.4%) compared to Holsteins steers at 55.7% (Forrest, 1981). The dressing percentages of both Charolais × Holstein steers and Hereford × Holstein steers ranged from 55% to 57% when fed to target end weights of 454, 544, and 635 kg (Fahmy and Lalande, 1975). Carcasses of Charolais × Holstein calves were 9 kg, 27 kg, and 33 kg heavier than those of Limousin × Holstein, Angus × Holstein, and purebred Holstein calves, respectively (Rezagholivand et al., 2021). In addition, INRA 95 × Holstein and Limousin × Holstein calves were heavier (29 kg and 24 kg, respectively) than Holsteins (Rezagholivand et al., 2021). The dressing percentages of INRA 95, Limousin, and Charolais-sired calves exceeded those of Angus-sired calves and purebred Holsteins by 4%–6% (Rezagholivand et al., 2021). Although data are limited regarding the translation of these dressing percentage difference to saleable red meat yield, it has been suggested that INRA 95, Limousin, and Charolais-sired calves out of Holstein dams yielded 1%–3% more saleable meat than Angus × Holstein or purebred Holstein calves, regardless of carcass weight (Rezagholivand et al., 2021).

Because the Jersey bull calf is undervalued compared to the Holstein, it is perhaps more important to note the shift crossbreeding has caused in the Jersey carcass characteristics when fed concentrate-based diets. The carcasses of Jersey steers were about 30 kg lighter than those of Angus × Jersey and SimAngus × Jersey steers, though their carcass weights did not differ from Red Wagyu × Jersey steers (Jaborek et al., 2019a). The average dressing percentage of purebred Jersey steers was 61.2%, which was less than the dressing percentage of Angus (64.2%), SimAngus (63.4%), and Red Wagyu-sired steers (63.2%) out of Jersey cows (Jaborek et al., 2019a). A similar evaluation of beef × Jersey animals reported that the carcasses of SimAngus and Angus-sired heifers and steers were about 20 kg heavier than Red Wagyu-sired heifers and steers (Jaborek et al., 2019b). Despite these differences in carcass weight and dressing percentage, there was no difference in the percentage of boneless closely trimmed retail cuts from Jersey, Angus × Jersey, SimAngus × Jersey, or Red Wagyu × Jersey carcasses (Jaborek et al., 2019a, 2019b).

While many instances cite that the incorporation of beef genetics increases the red meat yield of beef × dairy progeny raised for beef production, this impact is less clearly researched in the current body of literature. Regardless of the sire breed and finishing system, beef × dairy crossbreds appear to have an advantage in both carcass weight and dressing percentage when compared to purebred dairy cattle. It should be noted that many of the aforementioned dressing percentages remain less than those expected in native beef cattle, even for the beef × dairy progeny. Native beef cattle typically have a dressing percentage closer to 63%.

### Frame Size

One of the reasons that dressing percentages differ between dairy and beef breeds is that finished Holsteins are longer and taller than their native beef counterparts. We refer to this length and height collectively as “frame size”. One concern associated with processing Holstein beef is the larger frame size, relative to beef, because meat packing plants are not designed to accommodate long carcasses—measured as caudal to dorsal length on the rail—causing these carcasses to drag on the kill floor, in some extreme instances. Because heterosis effects have been observed for frame size, there are concerns that beef-sired dairy crossbred frame size may also negatively impact the packer (Bertrand et al., 1983). The Angus × Holstein terminal index includes the trait of yearling height to mitigate problems associated with frame size. Because the Jersey is a smaller framed cow, yearling height is not included in the Angus × Jersey terminal index. However, one experiment designed to measure heterosis of various traits among Angus, Brahman, Hereford, Holstein, and Jersey estimated the heterosis of carcass length was small (Baker et al., 1984). Thus, there remains some disagreement about the relevance of frame size, and relatively few scientific data to go by.

In one forage-finished trial, Holstein and Charolais × Holstein-Friesian bulls were harvested at a target weight of 650 kg and managed to 25 months of age in an Irish beef production system. Holstein bulls were 10 cm taller than crossbred bulls at the withers and 8 cm taller than crossbreds at the pelvis (McGee et al., 2007). The backs of live Holstein bulls measured 6 cm longer than those of Charolais × Holstein-Friesian bulls and the carcasses of Holstein bulls were subsequently 7 cm longer than Charolais × Holstein-Friesian bulls (McGee et al., 2007).

Other work from concentrated-based feeding systems seem to agree that dairy cattle are larger framed. In one experiment, the average Holstein carcass was 134 cm, which was longer than that of any other purebred animals or crossbreds (Baker et al., 1984). It should be noted that this trial included both beef sires and dams in a diallel cross; thus, in discussions they will simply be referred to as F1 crosses. Of the F1 Holstein crossbreds evaluated, those with a Hereford parent had the shortest carcasses (127 cm) and those with a Brahman parent had the longest (132 cm; Baker et al., 1984). When measured at a target final body weight, Holstein steers also had bodies 4 cm longer than Simmental × Holstein steers and 2 cm longer than Limousin × Holstein steers (Forrest, 1980, 1981). Similarly, Holstein steers finished in an intensive feedlot system were 1–2 cm longer than Angus × Holstein, Charolais × Holstein, Limousin × Holstein, and INRA 95 × Holstein steers (Rezagholivand et al., 2021). Between Charolais × Holstein steers and Hereford × Holstein steers, while Charolais-sired cattle were numerically 4 cm taller, sire breed did not impact back length (Fahmy and Lalande, 1975).

Generally, detectable differences in height and length exist between Holsteins and beef × Holstein crossbreds. Despite potential concerns of adding large Continental cattle genetics on already large Holsteins, the addition of Charolais genetics mitigated the undesirable Holstein measurements across forage and grain production systems. Many of the differences between breeds that were finished on concentrate-heavy diets were <5 cm, which may not be a great enough change to be relevant to the meat packer. Because larger differences existed between forage-finished cattle and most studies that have evaluated size are decades old, reevaluation of size traits in beef × Holstein crossbreds may be warranted. As previously mentioned, British breeds in the United States have been selected for greater body weights which has resulted in Angus exceeding continental breeds in direct breed effect for mature body weight (Zimmermann et al., 2021). Because Angus is currently the most common beef breed being mated to dairy animals in the United States, a comparison of the carcass lengths of U.S. Holstein steers with those of modern beef × Holstein steers may provide insight to what, if any, frame size selection parameters (i.e. yearling height) should be considered when breeding beef × Holstein.

### Ribeye Area

The area of the *longissimus dorsi* muscle cut at the 12th rib, known to the beef industry as the ribeye area (**REA**), can impact the value of a beef carcass in the United States because it pertains to Yield Grade. As ribeye area increases in proportion to carcass weight, the Yield Grade decreases. Because many of the studies pertaining to beef × dairy carcasses were performed internationally, direct measures of Yield Grade are not reported but various measures of the *longissimus* muscle often are.

#### Forage Finished.

In Europe, carcasses are typically quartered at the 10th rib rather than the 12th, the US standard. As such, the following reports of *longissimus* muscle area are reported based on measurements at the 10th rib (Kempster et al., 1982, 1988). The average REA of purebred British Friesian steers killed at 16 months of age was 56 cm^2^ while those killed at 24 months had REA of 53 cm^2^ (Kempster et al., 1982). When killed at 16 months, Charolais and Simmental-sired steers out of British Friesian dams had a 13 cm^2^ and 9 cm^2^ increase in REA over the Friesian steers while Angus and Devon-sired steers had REA 4 cm^2^ smaller the Friesian steers and those of Hereford-sired steers were 5 cm^2^ smaller (Kempster et al., 1982). When killed at 24 months, Charolais and Simmental-sired steers had 11 cm^2^ and 5 cm^2^ larger REA than British Friesian steers while Angus, Devon, and Hereford-sired steers had 9 cm^2^, 4 cm^2^, and 4 cm^2^ smaller REA than British Friesian steers, respectively (Kempster et al., 1982). The REA of Sussex × British Friesian steers did not differ from British Friesian steers (Kempster et al., 1982). A similar study reported that Charolais, Limousin, and Simmental-sired animals had REA 7–11 cm^2^ greater than those of British Friesian and Canadian Holstein cattle when killed at 16 months while animals sired by Hereford, Lincoln Red, South Devon, and Sussex bulls did not differ from dairy breeds in REA (Kempster et al., 1988). When slaughtered at 24 months, Charolais × Friesian and Limousin × Friesian steers exceed dairy steers in REA by 10–13 cm^2^ while steers sired by other beef breeds did not differ in REA from British Friesians or Holsteins (Kempster et al., 1988). In other publications, *longissimus* muscle was measured as a proportion of carcass weight. In such instances, all beef crossbreds had heavier *longissimus* muscles than dairy animals as they all had heavier carcasses (McGee et al., 2007; Huuskonen et al., 2013a, 2013b).

#### Concentrate Finished.

 The REA in the following studies was measured at the cross-section of the 12th rib. Similar to the reports of cattle reared in forage-based systems, F1 Hereford-Holstein and Angus-Holstein crossbreds did not differ from purebred Holsteins in REA in intensively managed, grain-based systems either (Baker et al., 1984). In addition, F1 Brahman-Holstein cattle exceeded purebred Holsteins in longissimus muscle area and F1 crosses of Jersey and Angus, Hereford, or Brahman exceeded purebred Jerseys in REA by 13 cm^2^, 14 cm^2^, and 17 cm^2^, respectively (Baker et al., 1984). Despite the advantage beef genetics added to Jersey ribeye area, beef × Jersey crossbreds did not differ from purebred Holsteins in longissimus muscle area (Baker et al., 1984). When comparing Angus, Hereford, and Charolais-sired cattle out of Brown Swiss dams there was no significant differences in REA between sire breed (Urick et al., 1974). However, when killed at 635 kg, REA from Charolais × Holstein cattle exceeded those from Herford × Holstein cattle by 14 cm^2^ (Fahmy and Lalande, 1975). The REA of Limousin × Holstein heifers and steers, Simmental heifers and steers, and Charolais steers exceeded Holstein steers by 17–18 cm^2^, 8 cm^2^, and 10 cm^2^, respectively (Forrest, 1977, 1980, 1981).

More modern data are limited in progeny from Holstein dams, in particular, but sire difference may still be more relevant to current genetics. Lucas et al. (2021) reported that LimFlex progeny out of Holstein or Jersey dams had smaller REA than Limousin progeny out of the same dam breeds (Lucas et al., 2021). When standardized to a common carcass weight, Jersey steers had similar ribeye areas to Angus × Jersey, SimAngus × Jersey, and Red Wagyu × Jersey steers (Jaborek et al., 2019a). Angus and SimAngus-sired steers had heavier carcasses than Jersey steers so their ribeyes were larger but did not impact the Yield Grades between sire breeds (Jaborek et al., 2019a). Similarly, no differences were detected in ribeye area when adjusted for carcass weight between Angus, SimAngus, and Red Wagyu-sired heifers and steers though Red Wagyu cattle had lighter carcasses (Jaborek et al., 2019b). Limousin × Holstein cattle had greater ribeye areas than Limousin × Jersey animals (Lucas et al., 2021).

With few exceptions, the previous beef × dairy data suggests that Continental beef breeds, like Charolais, Limousin, and Simmental, sire progeny with greater REA than their purebred dairy contemporaries while the British breeds, Angus and Hereford, sire progeny with the same or less REA than their purebred dairy contemporaries. Much of the data reported are likely a product of the era the bulk of the research addresses. After the 1980s, greater selection emphasis was placed on increasing the *longissimus* muscle area of beef cattle. Studies that measured the *longissimus* muscle as a proportion of carcass weight, or that corrected REA for carcass weight, detected smaller and fewer differences between dairy and beef × dairy breeds.

### Backfat

In the United States, it is common to report backfat in mm of thickness at the 12th rib interface. In this system, backfat measurements correspond with USDA Yield Grades, and thicker backfat is associated with a greater Yield Grade. However, in Europe, carcass fat is only visually assessed and assigned a EUROP fat score, but greater values also correspond with more subcutaneous fat cover.

In Scandinavian forage finished systems, beef-sired crossbred cattle from double-muscled breeds, like Blonde d’Aquitaine, typically had reduced fat scores than their purebred dairy counterparts while beef-sired progeny from British and Continental beef breeds typically had greater fat scores (Huuskonen et al., 2013a, 2013b). In concentrate finishing systems, data are more conflicted. Beef-sired cattle out of Brown Swiss cows all had similar backfat thickness (Urick et al., 1974). Similarly, no differences in backfat were observed between Jerseys, Holsteins, and crosses of those dairy breeds with Angus, Brahmans, and Herefords (Baker et al., 1984). However, Hereford × Holstein steers exceeded Charolais × Holstein steers in backfat thickness by 1.7 mm when slaughtered at 454 kg, 2.6 mm when slaughtered at 544 kg, and by 8.2 mm when slaughtered at 635 kg (Fahmy and Lalande, 1975). Backfat thickness was similar among carcasses from Charolais × Holstein heifers and steers and Simmental × Holstein steers had similar backfat thickness when compared to Holstein steers; however, carcasses from Simmental × Holstein heifers had backfat that was 1.6–4.6 mm thicker than all steers, crossbred or purebred Holstein (Forrest, 1977, 1980). Similarly, Limousin × Holstein steers did not differ from Holstein steers in backfat thickness, but Limousin × Holstein heifers had backfat 6.8 mm thicker than Holstein steers (Forrest, 1981). LimFlex × dairy cattle had thicker backfat than Limousin × dairy animals (Lucas et al., 2021).

Once again, Jersey influence has been less broadly studied; however, similar trends seem to emerge regarding backfat. Carcasses from Angus × Jersey steers had backfat that exceeded purebred Jersey steers by 5.1 mm; backfat of SimAngus × Jersey steers and Red Wagyu × Jersey steers were intermediate (Jaborek et al., 2019a). After adjusting backfat thickness measurements to a common carcass weight, a similar study determined combined average backfat thickness Angus × Jersey heifers and steers was 3.0 mm thicker than that of SimAngus × Jersey heifers and steers and 3.6 mm thicker than that of Red Wagyu × Jersey heifers and steers (Jaborek et al., 2019b).

The literature presented suggests that there is less variation in backfat thickness between dairy and beef × dairy breeds than in the other carcass traits discussed. Although heifers often exceeded steers in backfat thickness, this is a common trend even among native beef breeds. In most cases, Continental beef breeds sired progeny that produced carcasses with intermediate fat scores when compared to progeny from British breed beef sires, that produce carcasses with a great deal of fat, and progeny from purebred dairy or double-muscled sires that produce carcasses with very little fat.

### Marbling

When assessing carcasses harvested at less than 30 months of age, marbling score is used to determine the USDA Quality Grades. Of these grades, the 3 are most common among fed cattle are Prime, Choice, and Select. Marbling scores are assigned in the United States based on visual appraisal of nine depictions of intramuscular fat (**IMF**), the least fat is represented by the name “Practically Devoid”, while the most fat is termed “Abundant” and each of these depictions correlates to a USDA Quality Grade. Most studies convert IMF from a marbling score to a numeric scale, creating a continuous variable, where scores from 100 to 299 are uncommon in fed cattle, scores that range from 300 to 399 equate to Slight (representing the Select Grade in fed cattle), from 400 to 699 equate to Small to Moderate amounts of fat (representing a gradient withing the USDA Quality Grade of Choice), while abundant marbling would correspond to a Prime grade. The last iteration of these grades was published in 1996. Little data on IMF in forage-finished beef × dairy cattle exists because production systems finishing beef × dairy cattle on forage are predominantly international systems where IMF is not valued and there are not carcass measures that translate well to IMF (Liu et al., 2020). Cattle fed grain-based diets often have improved marbling scores compared to those finished on grass which is why most US production systems feed grain. For these reasons, all marbling data reported are from concentrate-finished cattle. Marbling units will be reported in relation to the numeric scale above, unless otherwise specified.

Baker et al. (1984) reported marbling on a scale of 5–15, where 5 was the least amount of marbling and 15 the most. The F1 Angus-Holstein, Brahman-Holstein, and Hereford-Holstein cattle did not achieve marbling scores significantly different from purebred Holstein or Jersey animals (Baker et al., 1984). Conversely, Jerseys crossed with Angus, Brahman, or Hereford had significantly higher marbling scores than purebred Jerseys (2.6-, 2.0-, and 3.2-point advantage, respectively) and Holsteins (3.0-, 2.4-, and 3.6-point advantage, respectively; Baker et al., 1984). No differences in marbling score were detected between Angus, Charolais, and Hereford-sired steers out of Brown Swiss dams (Urick et al., 1974). These data are difficult to correlate to the USDA Quality Grades because the Quality Grade system in place currently dates back only to 1996, despite the long history of the grading system in the United States.

More recent data may be more relevant to the current grading system and they report that, despite all purebred Jersey and Jersey crosses achieving a USDA Quality Grade of Choice, Angus × Jersey genetics increased the numeric marbling score when compared to purebred Jersey, SimAngus × Jersey, or Red Wagyu × Jersey (Jaborek et al., 2019a, 2019b). LimFlex-sired cattle out of Holsteins and Jerseys achieved greater marbling scores than Limousin-sired cattle out of Holsteins and Jerseys (Lucas et al., 2021).

Certain beef sire breeds, notably those with British influence, improved the marbling of beef from beef × dairy progeny at times. Limited data suggest that Continental beef sires may limit beef × dairy progeny marbling, but these data were excluded from this review as methodology on animal feeding was not reported (Gault et al., 2015). Differences in genetics and management systems make the older data reported difficult to apply to modern systems. The United States has been harvesting close to 85% Choice carcasses in more recent years. The influence of beef × dairy crossbreds on the prevalence of Choice carcasses in the US system is a topic that deserves more attention.

### Tenderness and Sensory Evaluations

Sensory panel evaluation and shear force testing provide data on eating quality of the beef. The shear force values reported in this review are reported as kg of force required to shear cooked beef.

There were no differences in tenderness, as determined by shear force, between Hereford, Hereford × Holstein, and Holstein carcasses finished on grass (Muir et al., 2000). The same was true of sheer force evaluation of beef from Hereford, Angus, and Charolais-sired steers out of Brown Swiss dams finished on concentrate-based diets (Urick et al., 1974). More modern genetics and feeding systems have emphasized eating quality. Cattle evaluated in these more modern systems indicate beef from SimAngus × Jersey (2.48 kg) and Red Wagyu × Jersey (2.39 kg) steers was more tender than beef from Angus × Jersey (2.76 kg) and purebred Jersey (2.71 kg) steers (Jaborek et al., 2019a). A later study of crossbred heifers and steers yielded similar results (Jaborek et al., 2019b). While sire breed influenced shear force tenderness in intensively fed beef × Jersey carcasses, these differences of less than 0.5 kg of force would likely not be detectable by consumers.

In agreement, sensory panel evaluations of aroma, tenderness, juiciness, flavor, and overall liking of beef from Hereford, Holstein, and Hereford × Holstein animals did not differ between breeds (Muir et al., 2000). In addition, changing cow breeds did not impact sensory evaluations as few differences in sensory panel evaluation were detected in beef from Angus × Brown Swiss, Hereford × Brown Swiss, and Charolais × Brown Swiss steers (Urick et al., 1974). Therefore, the available data, while limited, suggest that beef × dairy should produce the tender and delicious beef product that US consumers are accustomed to eating.

## CONCLUSIONS

International and historic data suggest that, regardless of feeding strategy, crossbred beef × dairy cattle often have greater ADG and are more efficient than purebred dairy steers. In many research trials, Continental-sired progeny often had greater growth and efficiency advantages over British-sired progeny. However, it is relevant to note that there are instances in the literature of crossbred heifers performing worse than purebred Holstein steers. The current body of literature also suggests that beef × dairy cattle often have an advantage over purebred dairy cattle in carcass weight and dressing percentage; there is less evidence that carcass weight and frame size need to be intensively selected *against* because beef genetic influence has consistently reduced traits related to frame size when compared to purebred Holsteins. There were fewer data and less agreement regarding ribeye area, backfat, marbling, tenderness, and eating quality, and these areas should be addressed by future beef × dairy research projects. Admittedly, the variation by age at slaughter make growth performance and carcass data challenging to interpret. The inconsistent findings among much of the research emphasizes the need for more data involving modern genetics and management systems. In addition, it must be acknowledged again that, while relative comparison may be valuable, the heavy international base for the data preclude their direct interpretation to US production systems.

When beef × dairy calves are valued at a premium above dairy calves, utilizing beef semen in the US dairy herd is economically advantageous. However, crossbred calves must maintain their value throughout the beef supply chain for the calf premiums to continue. To maintain supply chain value, intentional sire selection must occur at the dairy, but this is challenging because there are few sire breeds can be directly selected for economically relevant growth performance traits, such as ADG, DMI, and feed efficiency. Using the currently available across breed EPDs for growth (weaning weight and yearling weight) and carcass merit (REA, marbling, and carcass weight) should give dairy producers the ability to make informed selection decisions that will benefit the entire beef supply chain.

The evolution of beef × dairy cattle must include adoption of (a) genetic selection and (b) management strategies that will allow crossbred progeny to maintain market viability. Only then will the resulting beef × dairy calves be profitable at all points in the supply chain, not just marketed at a premium off the dairy.
